# Untargeted Metabolomic Profiling of Extracellular Vesicles Isolated from Human Seminal Plasma

**DOI:** 10.3390/biom14101211

**Published:** 2024-09-26

**Authors:** Manesh Kumar Panner Selvam, Partha K. Chandra, Zahra Bakhtiary, David W. Busija, Suresh C. Sikka

**Affiliations:** 1 Department of Urology, Tulane University School of Medicine, New Orleans, LA 70112, USA; zbakhtiary@tulane.edu (Z.B.); ssikka@tulane.edu (S.C.S.); 2 Department of Pharmacology, Tulane University School of Medicine, New Orleans, LA 70112, USA; pchandr1@tulane.edu (P.K.C.); dbusija@tulane.edu (D.W.B.); 3 Tulane Brain Institute, Tulane University, New Orleans, LA 70118, USA

**Keywords:** seminal extracellular vesicles, metabolomics, metabolites, bioinformatics, semen

## Abstract

Seminal extracellular vesicles (SemEVs) are repositories of biomolecules, including metabolites involved in the regulation of sperm function. The correlation between the metabolite profile of SemEVs and semen parameters, along with their role in regulating sperm function, is an unexplored area. This preliminary study evaluated the metabolomic content of SemEVs. Semen samples were obtained from 18 healthy men, and SemEVs were extracted from seminal plasma using the size exclusion chromatography qEV Gen 2–35 nm column coupled with an automatic fraction collector. The physical characterization of SemEVs was carried out with the ZetaView PMX-430-Z QUATT laser system. EV protein markers were detected using Western blot. In addition, these SemEVs were used for metabolomic profiling and functional bioinformatic analysis. The mean concentration of isolated SemEVs was 1.7 ± 1.1 × 10^11^/mL of seminal plasma, whereas SemEVs size and zeta potential were 129.5 ± 5.5 nm and −40.03 ± 3.99 mV, respectively. Western blot analysis confirmed the presence of EV specific markers such as CD81, ALIX, and TSG101. A total of 107 metabolites were identified using this untargeted metabolomic approach in SemEVs. Bioinformatics analysis further revealed that metabolites associated with tyrosine metabolism were highly enriched in these SemEVs. Ingenuity Pathway Analysis (IPA) also indicated that these metabolites present in SemEVs were involved in the regulation of the free radical scavenging pathway. Furthermore, our metabolomic results suggest that these SemEV-associated metabolites may play a pivotal role in the maintenance of seminal plasma redox homeostasis.

## 1. Introduction

Extracellular vesicles (EVs), encompassing apoptotic bodies (>1000 nm), microvesicles (100–1000 nm), and exosomes (30–200 nm), are categorized based on size and cellular origin [1]. EVs are produced and released through specific mechanisms that encapsulate a diverse array of cargo molecules and exert various biological effects [2]. Seminal EVs (SemEVs) play important roles in sperm function and fertility [3,4], interacting with spermatozoa and influencing their motility, capacitation (the process that enables sperm to fertilize an egg), and survival within the female reproductive tract [5,6,7].

SemEVs contain a variety of bioactive molecules, including proteins, lipids, nucleic acids, and signaling molecules [8,9], which are involved in processes such as sperm maturation and sperm motility [6]. Omics techniques, particularly proteomics and RNA sequencing (transcriptomics), are commonly used to analyze SemEVs’ molecular contents to understand their roles in reproductive biology and fertility [4,10,11]. Integrating omics data from SemEVs with omics datasets from sperm cells or female reproductive tissues can provide a comprehensive understanding of the reproductive process and identify potential biomarkers or therapeutic targets for reproductive health.

Metabolites are as important as proteins. Metabolites originate from various cellular processes, including glycolysis, fatty acid metabolism, amino acid metabolism, and nucleotide metabolism. Several analytical techniques, such as mass spectrometry (MS), nuclear magnetic resonance (NMR) spectroscopy, and liquid chromatography (LC) coupled with MS or NMR, are widely used to profile the metabolic signatures of EVs [12]. Metabolomic analysis of EVs provides insights into the metabolic phenotype of cells, tissue microenvironments, and physiological or pathological conditions, and includes biomarker discovery for disease diagnosis and prognosis, elucidation of EV-mediated metabolic signaling pathways, and understanding the role of EV metabolites in intercellular communication and disease pathogenesis.

Although several studies have reported the metabolite content of sperm and seminal plasma [13,14,15], there is a paucity of literature regarding the metabolomics of seminal EVs. Our study objective was to isolate SemEVs from human seminal plasma and identify the metabolomic profile of SemEVs using the LC-MS/MS untargeted approach.

## 2. Materials and Methods

### 2.1. Study Subjects and Semen Analysis

Semen samples from healthy men (*n* = 18) with a mean age of 37.5 years were used for SemEV isolation, characterization, and metabolomic analysis. Following 2–3 days of sexual abstinence, semen samples were collected by masturbation and allowed to liquefy for 20–30 min at 37 °C. Semen volume, sperm motility, and concentration were assessed according to the World Health Organization (WHO) 2021 criteria [16]. Semen samples were centrifuged at 300× *g* for 10 min at 37 °C to separate sperm from seminal plasma. Then, the seminal plasma was centrifuged at 12,000× *g* for 30 min at 4 °C to remove the spermatozoa residues and other debris. The clear seminal plasma was stored at −80 °C until EV isolation.

### 2.2. Extracellular Vesicle Isolation from Seminal Plasma Using Size-Exclusion Chromatography (SEC) qEV Columns

Seminal plasma samples were thawed at 37 °C for 20 min and centrifuged again at 12,000× *g* for 30 min to remove residual debris. The supernatants were successively passed through 0.22 µm filters. The filtrates were collected and equilibrated at room temperature for 30–45 min prior to SemEV isolation. The qEV Gen 2–35 nm column (Izon Science, Christchurch, New Zealand) was placed on the column holder. After column detection, the automatic fraction collector (AFC) was preset to collect a SemEV fraction of 1.5 mL and a buffer volume of 2.9 mL. The carousel, arranged with properly labeled 2 mL microcentrifuge tubes, was loaded onto the AFC, and a 15 mL column reservoir was placed on top of the column. After equilibrating the single qEV 35 nm columns with 17 mL of 1× PBS, 0.5 mL of filtered seminal plasma was applied on top of a qEV column. When the sample volume was absorbed by the column and reached the upper frit of the column, 6 mL of freshly prepared 0.22 µm filtered 1× PBS was added to the top of the column. After buffer volume collection, the required 1.5 mL of SemEV was collected. The obtained fraction was centrifuged at 120,000× *g* for 70 min at 4 °C to concentrate the SemEVs. Finally, the pellets were resuspended in 100 µL PBS.

### 2.3. Characterization of SemEVs by the ZetaView Particle Metrix System

The size, concentration, and zeta potential of the isolated SemEVs were measured using the ZetaView PMX-430-Z QUATT laser system 405/488/520/640 with a fixed cell assembly and the related ZetaView v8.05.16 SP3 software (Particle Metrix, Meerbusch, Germany) [17]. Briefly, the system was calibrated and aligned with diluted (1:250,000) 100 nm polystyrene standard polymer particles in an aqueous suspension (Applied Microspheres, Leusden, The Netherlands). Prior to measurement, the samples were equilibrated at room temperature for 20 min. To achieve particle counts suitable for the ZetaView Particle Metrix system, the samples were diluted to the necessary concentration (1:1000) in deionized–distilled water (ddH_2_O). All samples were examined under identical settings (room temperature between 20° and 25 °C, pH 7.0, sensitivity 80, shutter speed 100). Eleven positions were measured in each replicate, with each measurement performed in triplicate, and outlier positions excluded.

### 2.4. Characterization of SemEVs by Western Blot

SemEVs resuspended in 20 µL of PBS were lysed in 50 µL of radioimmunoprecipitation assay (RIPA) buffer supplemented with a complete (Roche) protease inhibitor for 1 h at 4 °C. Then, the mixture was centrifuged at 12,000× *g* for 30 min at 4 °C, and the clarified lysate was transferred into a new 1.5 mL tube. Protein quantification was performed using the Pierce BCA Protein Assay kit (Thermo Fisher Scientific, Waltham, MA, USA) according to the manufacturer’s instructions. An equal amount of protein per sample was separated using SDS-PAGE on gradient (4–20%) Mini-PROTEAN^®^ TGX^TM^ Precast Gels (Bio-Rad, Hercules, CA, USA) and transferred to the membrane using the Trans-Blot^®^ Turbo^TM^ Transfer System (Bio-Rad, Hercules, CA, USA). Membranes were blocked in 5% nonfat milk in Tris-buffered saline with Tween 20 (TBS-T) buffer for a minimum of 1 h at room temperature, then probed (or re-probed) with the primary antibody overnight at 4 °C. Then, membranes were washed 4 times (10 min each), with 1× TBST (Tris-buffered saline, 0.1% Tween 20) by shaking gently and were then incubated with horseradish peroxidase conjugated-secondary antibody for 1 h at room temperature. The list of primary and secondary antibodies used is presented in Table 1. Membranes were washed again 4 times with 1× TBST (10 min each), then treated with enhanced chemiluminescence kit-Pierce™ ECL Western Blotting Substrate (Thermo Scientific, Rockford, IL, USA) for 1 min. ECL-reacted blots were exposed to ChemiDoc (ChemiDoc ^TM^ MP Imaging System, Bio-Rad, Hercules, CA, USA) to detect chemiluminescence signals.

### 2.5. Untargeted Metabolomic Analysis of SemEVs

Metabolomic profiling of SemEVs isolated from human seminal plasma was carried out by Gigantest Laboratory (Gigantest^TM^, Baltimore, MD, USA). In general, metabolites were extracted from SemEVs (*n* = 18) utilizing 99% LC-MS grade acetonitrile with 1% formic acid. The samples were sonicated and then added to an Ostro protein precipitation and phospholipid removal plate. The metabolite solution was pushed through the sorbent filter of the Ostro plate using Waters positive pressure-96 processor and collected in an MS plate whereas the protein and phospholipids remained trapped in the sorbent Ostro plate filter. The metabolite solution was then dried to 150 µL for acquisition.

A Thermo Scientific IQX MS coupled with a Vanquish UHPLC system (Thermo Fisher Scientific, Waltham, MA, USA) was used to withdraw and inject 2 µL of the sample maintained at 4 °C for data acquisition. A reversed-phase chromatography method, employing a Discovery^®^ HSF5 reverse phase high-performance liquid chromatography column (Sigma, St. Louis, MO, USA), was utilized with a 15 min run time. The mobile phases consisted of 0.1% formic acid in MS-water (aqueous) or acetonitrile (organic) phases. To guarantee instrument sensitivity and data accuracy, a calibration procedure was performed before data acquisition. The final metabolite intensity data was obtained by integrating the area under each chromatographic peak. Finally, data analysis was performed using Laboratory Information Management System (LIMS) (Gigantest^TM^, Baltimore, MD, USA).

### 2.6. Bioinformatic Analysis

The metabolites identified in the SemEVs were subjected to functional annotation and enrichment analysis using a publicly available metabolomic analysis tool such as MetaboAnalyst 6.0 (https://www.metaboanalyst.ca/, accessed on 4 August 2024). The proprietary curated database IPA was used to analyze the involvement of metabolites in the molecular and cellular processes.

## 3. Results

**Figure 1:**
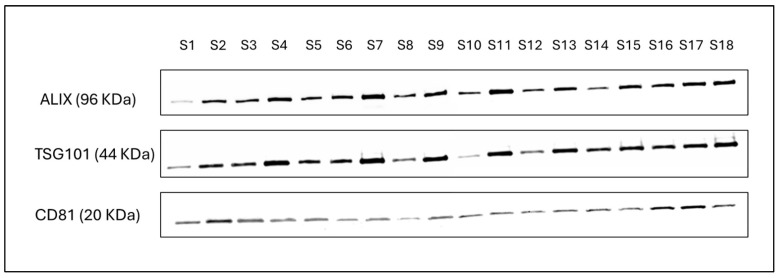
Detection of exosome specific markers such as CD81, ALIX, and TSG101 in SemEVs isolated from human semen. Original western blot images can be found in Supplementary File S1.

According to WHO 2021 criteria, mean semen parameters, such as volume, sperm concentration, and total sperm motility, were 1.8 ± 0.6 mL, 43.7 ± 9.3 × 10^6^/mL and 44.3 ± 10.9%, respectively. In addition, physical characterization revealed the mean particle concentration (1.7 ± 1.1 × 10^11^ particles/mL of seminal plasma), particle size (129.5 ± 5.5 nm), and zeta potential (−40.03 ± 3.99 mV) of EVs isolated from seminal plasma (Table 2). Western blot analysis indicated the presence of markers specific to exosomes, such as CD81, ALIX, and TSG101, in the SemEVs isolated using SEC qEV columns (Figure 1).

The untargeted metabolomic approach detected a total of 107 metabolites in the EVs isolated from each seminal plasma. These metabolites were mainly lipids, organic compounds, organic acids, etc. The metabolomic profile revealed 1-phenethylamine as the high metabolite present in the SemEVs. The top 10 high intensity metabolites are presented in Table 3.

**Figure 2:**
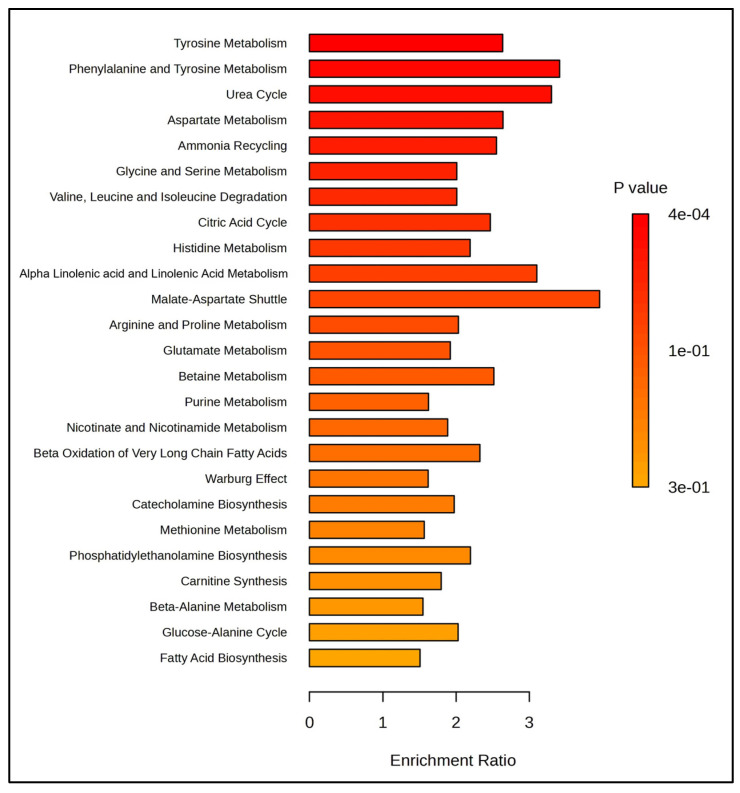
Pathways enriched with metabolites present in seminal extracellular vesicles. This figure was generated by MetaboAnalyst 6.0.

**Figure 3:**
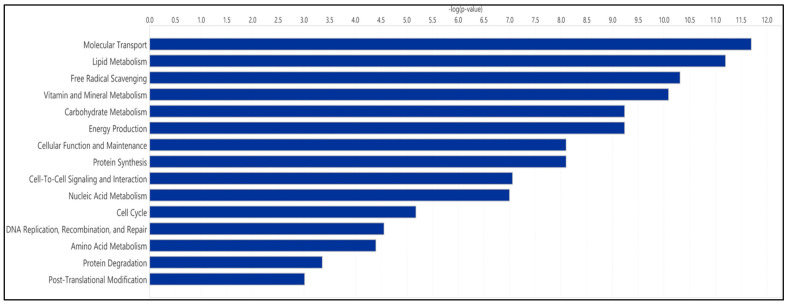
Molecular and cellular functions regulated by metabolites present in seminal extracellular vesicles. This figure was developed by IPA.

Enrichment analysis revealed that metabolites present in the SemEVs were associated with pathways such as tyrosine, phenylalanine, aspartate, glycine, and serine metabolism (Figure 2). Also, IPA indicated free radical scavenging as a significant molecular and cellular functions regulated by the metabolites (*n* = 30) present in the SemEVs (Figure 3, Table 4 and Table S1).

## 4. Discussion

Human semen contains EVs (SemEVs: exosomes and microvesicles) derived from various cells of the male reproductive system, including the seminal vesicles, epididymis, and prostate [4]. Physical and molecular characterization of biomolecules isolated using SEC columns confirmed the presence of SemEVs isolated from semen. SemEVs contain a variety of bioactive molecules, including proteins, lipids, nucleic acids, and signaling molecules. These biomolecules are involved in processes such as sperm maturation and sperm motility [3]. Metabolites are as important as other biomolecules in modulating sperm function [13,14,15]. In this study, we have reported the presence of 107 metabolites in the SemEVs using an untargeted metabolomic approach. One limiting factor of this study was the sample size. Including more samples might increase the number of metabolites identified using a global metabolomic approach.

EVs possess a diverse array of metabolites, reflecting the parent cells metabolic status, which originate from various cellular processes [18]. In sperm, the tyrosine metabolism pathway plays an important role in regulating functions such as capacitation, the acrosome reaction, and fertilization [19]. In addition, tyrosine phosphorylation is linked with hyperactivation, which is crucial for the ability of sperm to penetrate the oocyte zona pellucida [20,21]. Our bioinformatic analysis revealed that these SemEVs were enriched with metabolites involved in tyrosine metabolism, suggesting that the transfer of metabolite content from SemEVs to sperm during the maturation process is crucial for maintaining normal physiological function.

In general, EVs contain metabolites that play a major role in the redox signaling pathway. In semen, a physiological balance between oxidants (free radicals) and antioxidants (reductants) is necessary to maintain redox homeostasis [22]. Disturbance to the free radical scavenging system can result in oxidative stress and has adverse effects on normal sperm function [22,23,24]. The metabolomic profile in our study showed that metabolites involved in regulating the free radical scavenging system were enriched in SemEVs, which can counteract oxidative stress-induced sperm dysfunction. Excessive oxidative stress can result in sperm DNA fragmentation, which can cause fertilization and implantation failure [25,26]. Therefore, the metabolomic composition of SemEVs is crucial for controlling the damaging effects of oxidative stress on sperm function.

## 5. Conclusions

This study reports the metabolomic content of EVs isolated from human seminal plasma. Our untargeted approach confirmed the presence of metabolites in SemEVs linked with sperm function and oxidative stress. This pilot study lays the foundation for further research on the diagnostic and therapeutic role of SemEV metabolites in male infertility. In addition, evaluating the functional role of these key metabolites in SemEVs will enable biomarker identification to predict reproductive outcomes in the assisted reproductive technology procedures.

## Figures and Tables

**Table 1:** List of primary and secondary antibodies used in this study.

Primary				Secondary		
Protein	Antibody	Manufacturer	Dilution	Antibody	Manufacturer	Dilution
ALIX	Monoclonal Anti-Human Mouse IgG	Cell Signaling Technology (Danvers, MA, USA) Alix (3A9)	1:5000	HRP conjugated Anti-Mouse Goat IgG	Abclonal (Woburn, MA, USA) AS003	1:5000
CD81	Monoclonal Anti-Human Rabbit IgG	Abclonal A23455	1:10,000	HRP conjugated Anti-Rabbit Goat IgG	Abclonal AS014	1:10,000
TSG101	Polyclonal Anti-Human Rabbit IgG	Abclonal A1692	1:1000			

ALIX: ALG-2-interacting Protein X; CD81: Cluster of Differentiation 81; TSG101: Tumor susceptibility gene 101.

**Table 2:** Physical characteristics of seminal extracellular vesicles isolated from human semen using size-exclusion chromatography qEV columns.

Sample ID	Conc. (SemEV/mL of Seminal Plasma)	Size (nm)	Zeta Potential (mV)
S1	4.2 × 10^10^	145.5	−37.24
S2	3.9 × 10^11^	130.9	−37.66
S3	2.7 × 10^11^	125.1	−39.60
S4	3.9 × 10^11^	120.2	−40.88
S5	5.1 × 10^10^	134.4	−30.66
S6	1.4 × 10^11^	130.7	−39.09
S7	6.9 × 10^10^	126.2	−37.74
S8	2.7 × 10^11^	128.6	−44.03
S9	1.1 × 10^11^	125.2	−33.30
S10	2.2 × 10^11^	130.6	−45.18
S11	1.3 × 10^11^	132.8	−42.22
S12	9.6 × 10^10^	124.6	−41.96
S13	4.8 × 10^10^	131.5	−40.28
S14	1.3 × 10^11^	125.9	−41.25
S15	9.0 × 10^10^	130.7	−46.23
S16	2.3 × 10^11^	130.5	−44.92
S17	2.4 × 10^11^	124.6	−38.82
S18	1.5 × 10^11^	132.7	−39.42

**Table 3:** The top metabolites identified in the extracellular vesicles isolated from seminal plasma.

Metabolites	HMDB ID	Average Intensity
High Abundant		
1-phenethylamine	HMDB0002017	10.3 ± 0.38 × 10^7^
gamma-linolenic acid	HMDB0003073	9.03 ± 0.81 × 10^7^
linolenic acid	HMDB0001388	9.03 ± 0.81 × 10^7^
linoleic acid	HMDB0000673	8.65 ± 0.81 × 10^7^
oleic acid	HMDB0000207	7.51 ± 0.71 × 10^7^
aminoacetone	HMDB0002134	6.65 ± 0.57 × 10^7^
9Z-hexadecenoic acid	HMDB0003229	3.87 ± 0.35 × 10^7^
L-leucine	HMDB0000687	3.03 ± 2.12 × 10^7^
L-isoleucine	HMDB0000172	3.03 ± 2.12 × 10^7^
cinnamic acid	HMDB0000567	2.14 ± 0.08 × 10^7^

**Table 4:** Seminal extracellular vesicle metabolites involved in the regulation of the free radical scavenging pathway.

Function	Number of Metabolites	Metabolites
Free Radical Scavenging	30	2-oxoglutaric acid, 3-hydroxyanthranilic acid, 5-hydroxytryptamine, adenosine, beta-estradiol, betaine, choline, citric acid, citrulline, creatine, dopamine, gamma-linolenic acid, glucosamine-6-phosphate, hexanoic acid, L-carnitine, L-glutamic acid, L-methionine, L-tryptophan, linoleic acid, norepinephrine, octanoic acid, oleic acid, palmitic acid, stearic acid, succinic acid, tyramine, uric acid, uridine, vitamin A, xanthine

## Data Availability

The study data are available from the corresponding author upon reasonable request.

## Supplementary Materials

The following supporting information can be downloaded at: https://www.mdpi.com/article/10.3390/biom14101211/s1, Table S1: Molecular and cellular functions regulated by metabolites present in SemEVs; File S1: original western blot images.
