# Supplementary material for: Untargeted Metabolomic Profiling of Extracellular Vesicles Isolated from Human Seminal Plasma
Source: Biomolecules. 2024 Sep 26;14(10):1211. doi: 10.3390/biom14101211 (PMC11506783; doi:10.3390/biom14101211)
Supplement: Supplementary file 1 [file biomolecules-14-01211-s001.zip › biomolecules-3169184-supplementary table S1.pdf]

Table S1: Molecular and cellular functions regulated by metabolites present in SemEVs

Functions	# of Metabolites	Metabolites
Molecular Transport	51	1-palmitoyl-lysophosphatidic acid,2-oxoglutaric acid,3-hydroxyanthranilic acid,5-hydroxytryptamine,9Z-hexadecenoic acid,adenosine,alpha-keto-beta-methylvaleric acid,alpha-ketoisocaproic acid,beta-estradiol,betaine,choline,citric acid,citrulline,creatine,dopamine,eicosa-11Z, 14Z-dienoic acid,gamma-glutamylcysteine,gamma-linolenic acid,heptadecanoic acid,heptanoic acid,hexanoic acid,hypoxanthine,L-aspartic acid,L-carnitine,L-glutamic acid,L-glutamine,L-leucine,L-methionine,L-phenylalanine,L-serine,L-threonine,L-tryptophan,L-tyrosine,linoleic acid,linolenic acid,myristic acid,N(1)-methylnicotinamide,norepinephrine,octanoic acid,oleic acid,orotic acid,palmitic acid,phenylpyruvic acid,pyridoxine,stearic acid,succinic acid,tyramine,uric acid,uridine,vitamin A,xanthine
Lipid Metabolism	41	2-oxoglutaric acid,3-hydroxyanthranilic acid,5-hydroxytryptamine,adenosine,alpha-ketoisocaproic acid,beta-estradiol,betaine,choline,citric acid,citrulline,creatine,D-alpha-hydroxyglutarate,dopamine,fumaric acid,hexanoic acid,hypoxanthine,isocitric acid,L-carnitine,L-glutamic acid,L-glutamine,L-methionine,L-phenylalanine,L-serine,L-threonine,linoleic acid,linolenic acid,myristic acid,N(1)-methylnicotinamide,norepinephrine,octanoic acid,oleic acid,orotic acid,palmitic acid,phenol,phosphorylcholine,pyridoxine,stearic acid,succinic acid,uric acid,uridine,vitamin A
Free Radical Scavenging	30	2-oxoglutaric acid,3-hydroxyanthranilic acid,5-hydroxytryptamine,adenosine,beta-estradiol,betaine,choline,citric acid,citrulline,creatine,dopamine,gamma-linolenic acid,glucosamine-6-phosphate,hexanoic acid,L-carnitine,L-glutamic acid,L-methionine,L-tryptophan,linoleic acid,norepinephrine,octanoic acid,oleic acid,palmitic acid,stearic acid,succinic acid,tyramine,uric acid,uridine,vitamin A,xanthine

Vitamin and Mineral Metabolism	32	1-palmitoyl-lysophosphatidic acid,5-hydroxytryptamine,9Z-hexadecenoic acid,adenosine,alpha-ketoisocaproic acid,beta-estradiol,choline,citric acid,creatine,dopamine,eicosa-11Z, 14Z-dienoic acid,gamma-glutamylcysteine,gamma-linolenic acid,heptadecanoic acid,heptanoic acid,hexanoic acid,hypoxanthine,L-glutamic acid,L-phenylalanine,L-tryptophan,linoleic acid,linolenic acid,myristic acid,norepinephrine,octanoic acid,oleic acid,palmitic acid,stearic acid,succinic acid,uric acid,vitamin A,xanthine
Carbohydrate Metabolism	36	3,4-dihydroxyphenylacetic acid,3-hydroxyanthranilic acid,5-hydroxytryptamine,adenosine,alpha-keto-beta-methylvaleric acid,alpha-ketoisocaproic acid,beta-estradiol,betaine,choline,citric acid,citrulline,creatine,D-alpha-hydroxyglutarate,hexanoic acid,L-glutamic acid,L-glutamine,L-methionine,L-phenylalanine,L-serine,L-threonine,L-tryptophan,L-tyrosine,linoleic acid,myristic acid,norepinephrine,octanoic acid,oleic acid,orotic acid,palmitic acid,phenylpyruvic acid,phosphorylcholine,stearic acid,succinic acid,tyramine,uric acid,uridine
Energy Production	27	2-oxoglutaric acid,adenosine,alpha-keto-beta-methylvaleric acid,alpha-ketoisocaproic acid,beta-estradiol,choline,citric acid,creatine,dopamine,hexanoic acid,hypoxanthine,isocitric acid,L-aspartic acid,L-carnitine,L-glutamic acid,L-methionine,linoleic acid,myristic acid,norepinephrine,octanoic acid,oleic acid,palmitic acid,stearic acid,succinic acid,tyramine,uric acid,vitamin A
Cellular Function and Maintenance	35	2-oxoglutaric acid,3-hydroxyanthranilic acid,5-hydroxytryptamine,adenosine,alpha-ketoisocaproic acid,beta-estradiol,beta-hydroxyisovaleric acid,betaine,choline,citrulline,creatine,D-alpha-hydroxyglutarate,dopamine,hypoxanthine,L-aspartic acid,L-carnitine,L-glutamic acid,L-glutamine,L-methionine,L-phenylalanine,L-serine,L-threonine,L-tryptophan,L-tyrosine,linoleic acid,myristic acid,norepinephrine,oleic acid,palmitic acid,stearic acid,succinic acid,tyramine,uric acid,vitamin A,xanthine
Protein Synthesis	18	3-hydroxyanthranilic acid,5-hydroxytryptamine,beta-estradiol,beta-hydroxyisovaleric acid,citrulline,creatine,L-aspartic acid,L-carnitine,L-glutamic acid,L-methionine,L-phenylalanine,L-serine,L-tryptophan,linoleic acid,norepinephrine,oleic acid,palmitic acid,uric acid

Cell-To-Cell Signaling and Interaction	32	3-hydroxyanthranilic acid,5-hydroxytryptamine,adenosine,beta-estradiol,betaine,choline,citric acid,creatine,dihydroxyphenylethylene glycol,dopamine,hypoxanthine,L-aspartic acid,L-carnitine,L-glutamic acid,L-glutamine,L-methionine,L-phenylalanine,L-serine,L-tryptophan,L-tyrosine,linoleic acid,linolenic acid,myristic acid,norepinephrine,oleic acid,palmitic acid,phosphorylcholine,pyridoxine,stearic acid,tyramine,uric acid,xanthine
Nucleic Acid Metabolism	29	1-palmitoyl-lysophosphatidic acid,2-oxoglutaric acid,5-hydroxytryptamine,adenosine,alpha-ketoisocaproic acid,beta-estradiol,betaine,choline,citric acid,citrulline,creatine,dopamine,hypoxanthine,isocitric acid,L-aspartic acid,L-carnitine,L-glutamic acid,L-methionine,linoleic acid,myristic acid,norepinephrine,octanoic acid,oleic acid,palmitic acid,stearic acid,succinic acid,tyramine,uric acid,vitamin A
Cell Cycle	20	2-oxoglutaric acid,3-hydroxyanthranilic acid,adenosine,beta-estradiol,choline,citric acid,dopamine,L-aspartic acid,L-carnitine,L-glutamic acid,L-methionine,L-serine,L-tryptophan,linoleic acid,linolenic acid,norepinephrine,oleic acid,palmitic acid,stearic acid,uridine
DNA Replication, Recombination, and Repair	19	5-hydroxytryptamine,adenosine,beta-estradiol,citrulline,cytosine,dopamine,L-glutamic acid,L-methionine,L-phenylalanine,linoleic acid,myristic acid,norepinephrine,oleic acid,palmitic acid,phosphorylcholine,stearic acid,uric acid,uridine,vitamin A
Amino Acid Metabolism	19	2-oxoglutaric acid,5-hydroxytryptamine,adenosine,beta-estradiol,betaine,choline,citrulline,creatine,dopamine,L-aspartic acid,L-glutamic acid,L-leucine,L-methionine,L-phenylalanine,L-serine,L-threonine,L-tryptophan,L-tyrosine,pyridoxine
Protein Degradation	7	3-hydroxyanthranilic acid,beta-estradiol,beta-hydroxyisovaleric acid,L-carnitine,L-phenylalanine,oleic acid,palmitic acid

Post-Translational Modification	4	beta-estradiol,L-carnitine,L-tyrosine,uric acid
