# Supplementary figures and images for: Untargeted Metabolomic Profiling of Extracellular Vesicles Isolated from Human Seminal Plasma
Source: Biomolecules. 2024 Sep 26;14(10):1211. doi: 10.3390/biom14101211 (PMC11506783; doi:10.3390/biom14101211)

## Western Blot Images

**ALIX (96 KD):**

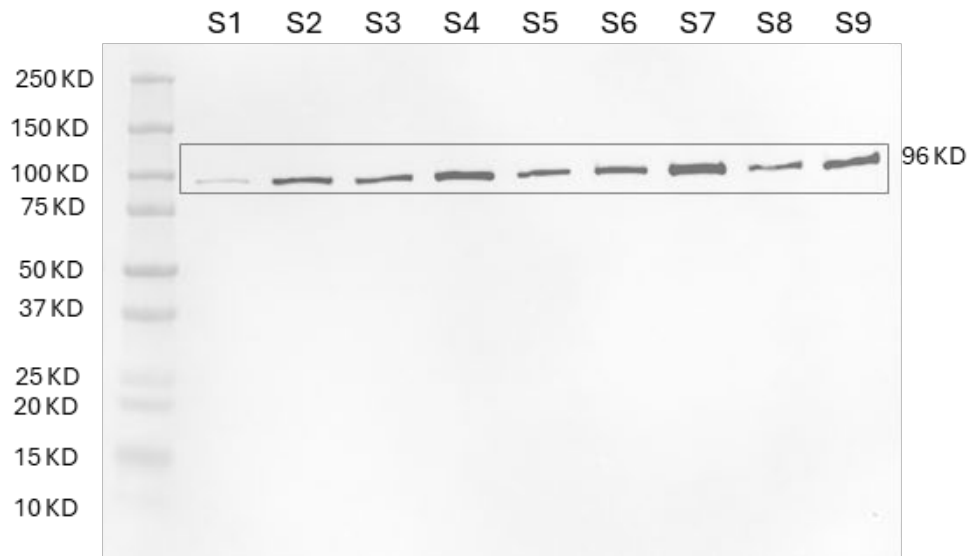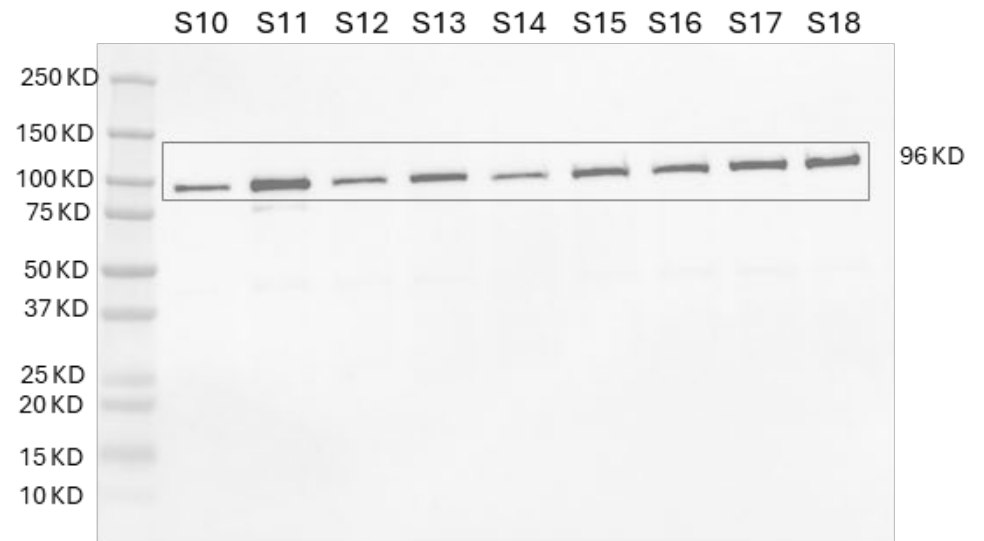

TSG 101 (44 KD):

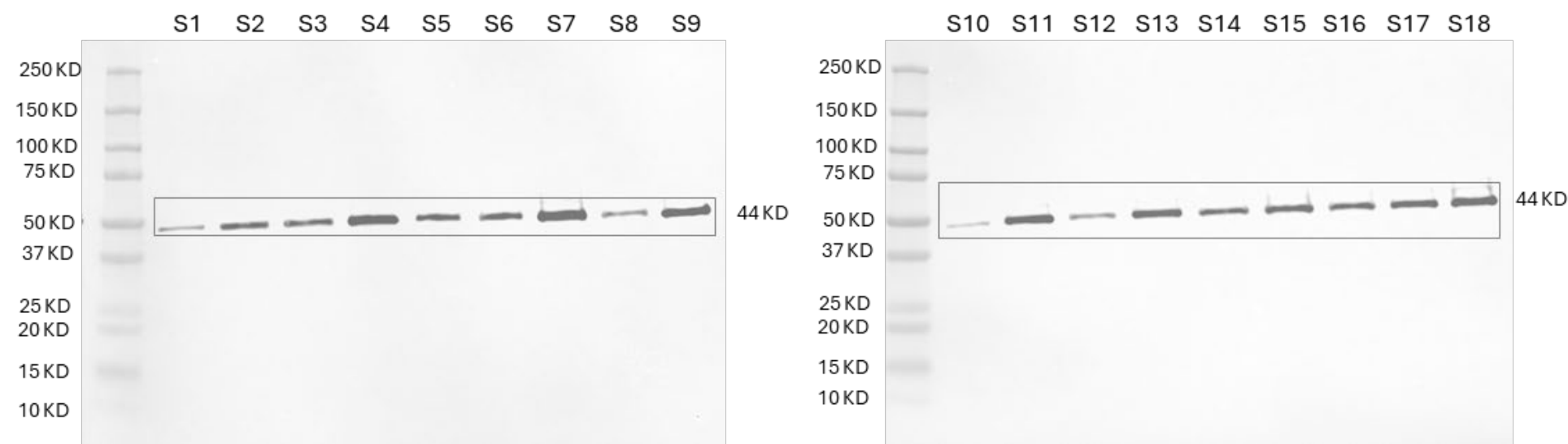

**CD81 (20 KD):**

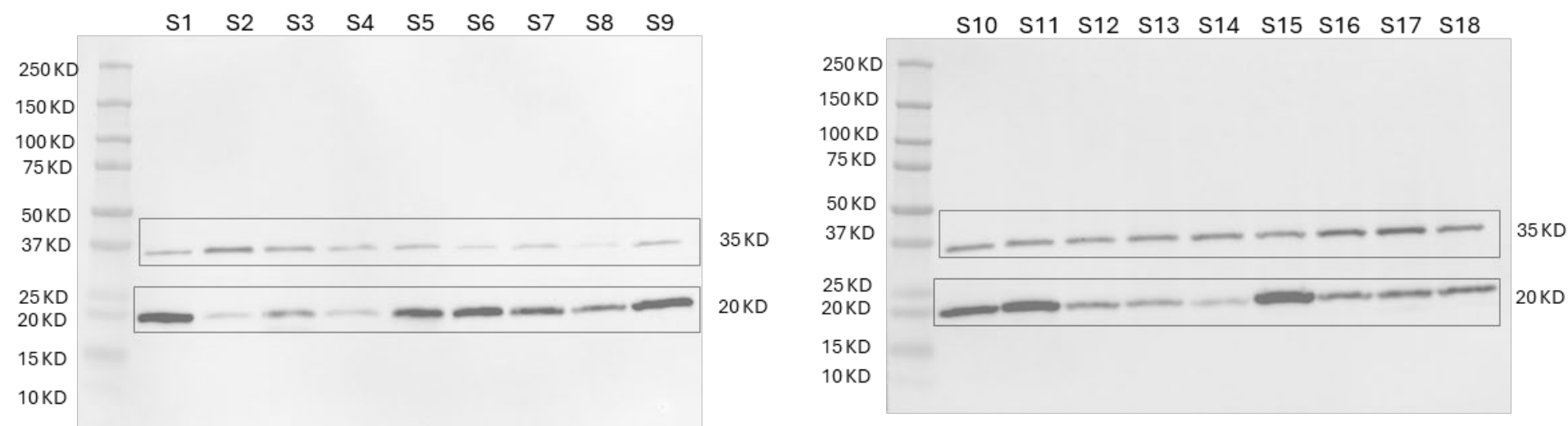

Supplement: Supplementary file 1 [file biomolecules-14-01211-s001.zip › biomolecules-3169184-Supplementary File S1 - WB images.pdf]
